# Psychometric properties of the Kogan Attitudes toward Older People Scale: evidence from a German general-population survey

**DOI:** 10.3389/fpsyg.2026.1916164

**Published:** 2026-08-25

**Authors:** Birgit Teichmann, Fany Pegiou, Shengze Zhi, Burcu Akpinar Söylemez

**Affiliations:** 1 Network Aging Research, Heidelberg University, Heidelberg, Germany; 2 Nursing Department, International Hellenic University, Thessaloniki, Greece; 3 School of Nursing, Jilin University, Changchun, Jilin, China; 4 Department of Internal Medicine Nursing, Faculty of Nursing, Dokuz Eylul University, Inciralti, Izmir, Türkiye

**Keywords:** age stereotypes, ageism, attitude, gerontology, psychometrics, reliability, validity

## Abstract

**Introduction:**

The Kogan Attitudes toward Older People Scale (KAOP) has been translated and validated in numerous languages, although a German version does not yet exist. The present study aimed to translate, adapt, and psychometrically evaluate the KAOP for German-speaking populations.

**Methods:**

A convenience sample of 419 participants completed the 34-item German KAOP Scale (KAOP-D-34), 211 of whom took part in the retest after four weeks. Reliability was assessed using internal consistency, item analysis, and test–retest reliability. Structural validity was explored using principal component analysis (PCA), and construct validity was tested using the known-groups method. Convergent validity was assessed utilizing the Aging Semantic Differential (ASD), Age Stereotypes (AS), and Future Selves (FS).

**Results:**

The KAOP-D-34 demonstrated good internal consistency (*α* = 0.870, *ω* = 0.863) and good test–retest reliability (ICC = 0.846, 95% CI: 0.802; 0.880). Items 7 and 8 were excluded due to poor content validity, increasing the reliability of the final 32-item scale (α = 0.876, ω = 0.867). PCA revealed a two-factor solution accounting for 30% of the total variance. Known-groups analyses showed significant differences in KAOP-D-32 scores according to gender, age group, educational level, and self-reported attitudes toward older adults. The final KAOP-D-32 total scores showed a significant correlation with the ASD (*r* = −0.40). Most AS and FS domains correlated significantly with KAOP-D32, except finances. Furthermore, higher KAOP-D-32 scores were associated with older age, better self-rated health, and greater life satisfaction.

**Conclusion:**

These results support that the KAOP-D-32 is a psychometrically robust instrument for assessing attitudes toward older adults.

## 1 Introduction

The rapid growth of the older adult population is one of the most significant demographic changes of the twenty-first century. It is reshaping healthcare delivery, social policy, and cultural narratives about later life (Khan et al., 2024). Crucially, aging today is not the same phenomenon that characterized previous generations. Longitudinal and comparative-cohort studies consistently show that later-born groups of older adults have significantly higher levels of physical, cognitive, and social functioning than earlier groups at the same chronological age (Beard et al., 2025; Kekäläinen et al., 2024). Consequently, the perception of when “old age” begins has also changed over the course of history. Compared to the earliest birth cohorts, later birth cohorts reported a later perceived onset of old age (Wettstein et al., 2024).

Contemporary gerontological theory emphasizes that aging is a multidimensional construct encompassing chronological age, biological age, and subjective age, which often diverge substantially (Salignon et al., 2023). The cohort-based improvement in health and functioning has been conceptualized as an extension of the “gained healthy life-years,” reflecting advances in medicine, public health, and living conditions (Reuter et al., 2023; Sauerberg et al., 2020). Recent research on biological aging further supports this notion, demonstrating that biological age—as indexed by physiological and molecular markers—has declined relative to chronological age across cohorts, indicating that later-born cohorts are biologically younger at the same chronological age than earlier ones (Ahrenfeldt et al., 2018). Subjective age—how old individuals feel—has repeatedly been shown to predict health, well-being, and mortality more strongly than chronological age, highlighting the relevance of internalized perceptions of aging (Kornadt et al., 2020; Kornadt and Rothermund, 2012). Various instruments have been developed to empirically assess attitudes or views on aging (Klusmann et al., 2020). Views on aging are defined as a person’s perceptions about older people, old age, and aging in general, as well as their perceptions of their own age and aging (Wurm et al., 2017). Early measures, such as the Riegel Scale (Riegel and Riegel, 1960) and Palmore’s Facts on Aging Quiz (Palmore, 1981), primarily conceptualized attitudes toward aging as a unidimensional construct emphasizing age-related decline or factual knowledge about aging. Subsequent theoretical developments, particularly those informed by the lifespan perspective (Baltes, 1987), led to the development of multidimensional instruments that acknowledge both age-related gains and losses.

Instruments differ substantially with respect to the construct they assess. Measures such as the Aging Semantic Differential (ASD; Rosencranz and McNevin, 1969) evaluate general attitudes toward older adults using bipolar adjective pairs (e.g., active/passive, flexible/rigid) and primarily capture affective and evaluative components of age-related attitudes. In contrast, more recent instruments assess individuals’ own views on aging or self-perceptions of aging, emphasizing the subjective experience of growing older across multiple life domains (Wurm et al., 2017). Domain-specific measures of age stereotypes further conceptualize aging stereotypes as multidimensional and context-dependent, varying across domains such as health, family, work, personality, and leisure (Kornadt and Rothermund, 2011; Kornadt et al., 2017, 2018). These instruments acknowledge that age stereotypes may simultaneously include positive and negative expectations depending on the life domain and the perspective assessed.

Recent reviews have highlighted the considerable expansion and diversification of measures assessing attitudes toward aging, self-perceptions of aging, and self-directed age stereotypes, while also demonstrating that available instruments differ in their conceptual focus, target population, dimensionality, and psychometric properties (Burton et al., 2021; Faudzi et al., 2019). Notably, many of these newer measures were developed specifically to assess self-perceptions of aging or self-directed age stereotypes among younger or older adults, rather than general attitudes toward older adults as a social group.

Despite these conceptual advances and the availability of more specific multidimensional measures, global attitude measures remain particularly valuable for assessing general attitudes toward older adults and facilitating comparisons across cultural contexts. Attitudes toward older adults as a social group remain a central topic of gerontological research, particularly in studies examining ageism and cross-cultural differences. Kogan’s Attitudes toward Older People Scale (KAOP) (Kogan, 1961) is a well-established instrument for assessing general attitudes toward older adults. In contrast to more recently developed instruments that primarily assess self-perceptions of aging, self-directed age stereotypes, or domain-specific expectations regarding aging, the KAOP captures broad evaluative attitudes toward older adults as a social group. Through positively and negatively worded statements, it assesses both favorable and unfavorable perceptions independent of respondents’ age or gender, providing a balanced measure of explicit age-related attitudes.

The KAOP has been translated and psychometrically evaluated in numerous cultural contexts, making it a valuable instrument for cross-cultural research. Its continued use over more than six decades reflects its relevance for investigating societal attitudes toward older adults and enables comparisons with a large body of international literature. At the same time, previous validation studies have reported inconsistent findings regarding its factorial structure, with one-, two-, and multi-factor solutions being proposed (Gansukh et al., 2025; Lambrinou et al., 2005; Ortiz-Rubio et al., 2020; Söderhamn et al., 2000). These findings underscore the importance of evaluating the instrument’s psychometric properties whenever it is adapted to a new language or cultural context.

To date, however, no validated German version of the KAOP is available, despite the existence of sophisticated German-language instruments assessing domain-specific age stereotypes and views on one’s own aging. As these instruments address conceptually distinct constructs, they do not provide a direct assessment of general attitudes toward older adults as a social group. A validated German version of the KAOP would therefore complement the existing measurement repertoire by enabling the assessment of explicit societal attitudes toward older adults and facilitating comparisons with international research. Such comparisons are particularly relevant for examining whether societal attitudes toward older adults have changed alongside improvements in health, longevity, and functioning in later life.

Against this background, the present study aimed to translate the KAOP into German and to comprehensively evaluate its psychometric properties in a German-speaking sample. Specifically, we examined its factorial structure, reliability, and construct validity to determine whether the German version provides a valid and reliable measure of general attitudes toward older adults and can be used in future national and cross-cultural research.

## 2 Methods

### 2.1 Study design

The study was designed as a cross-sectional survey with a second data collection of a subgroup to assess the retest reliability after 4 weeks. The present study followed the EQUATOR guidelines for reporting research using the Strengthening the Reporting of Observational Studies in Epidemiology (STROBE) checklist (von Elm et al., 2007) (Supplementary material 1).

### 2.2 Participants and data collection

A convenience sample of *N* = 419 was recruited through the Network Aging Research newsletters, as well as by sharing invitations to participate via social media platforms such as Facebook, WhatsApp, Instagram, and using the snowball sampling method. The inclusion criteria were limited to individuals aged 18 years or older whose native language was German. The online survey was conducted with SoSci Survey (Leiner, 2025). The data were collected between 17 December 2025 and 08 February 2026 at https://www.soscisurvey.de/Kogan/.

To ensure sufficient statistical power, we conducted an *a priori* power analysis using the G*Power 3.1.9.7 software program (Faul et al., 2007), following the procedure described by Kang (2021). G*Power recommended a total sample size of *N* = 244 for the Wilcoxon-Mann–Whitney tests to achieve the desired power level (1 − *β* = 0.95; d = 0.5). The allocation ratio was set to 3, anticipating a sample that is slightly overeducated. However, questionnaire-based studies typically suggest a sample size of 150–300 participants, or 5–20 participants per item (Polit and Beck, 2008; Memon et al., 2020). As the KAOP comprises 34 items, the final sample size of *N* = 419 exceeded these suggestions. In addition, a smaller subsample (*n* = 211) completed a shortened version of the survey again after a four-week interval, in order to assess the test–retest reliability of the KAOP Scale.

### 2.3 Questionnaire design

Participants provided information about their demographics, their experiences with elderly people, along with their answers to the ΚAOP Scale (Kogan, 1961), the ASD (Rosencranz and McNevin, 1969), the brief version of the domain-specific age stereotype and future self-views scales (Kornadt et al., 2020), and the Big Five Inventory (BFI-10) (Rammstedt, 2007). The order of the scales was predetermined, whereas the items within each scale were presented in randomized order. The questionnaire was estimated to require approximately 20–30 min to complete. We provide the German version of the scales as well as the data of the study on Open Science Framework (https://osf.io/8sae5/overview).

#### 2.3.1 Kogan Attitudes toward Older People (KAOP) Scale

The KAOP was developed in 1961 by Nathan Kogan to assess affective attitudes toward older adults (Kogan, 1961). The scale consists of two sets of items containing matched positive and negative statements about the housing arrangements of older adults, their competency, personal appearance, personality, their interpersonal relationships with other age groups, their dependence on others, and their socioeconomic power. This results in 17 negative sentiment items (N) and 17 positive sentiment items (P). Responses to the items are rated on a 6-point Likert scale, ranging from 1 (*strongly disagree*) to 6 (*strongly agree*). Negative statements were reverse-coded, resulting in a total score ranging from 34 to 204, where higher scores indicate a more positive attitude. The KOAP has already been validated in several languages with reported internal consistency coefficients (Cronbach’s alpha) ranging from 0.62 in the French version (Wanko Keutchafo and Kerr, 2024) to 0.89 in Arabic (Alquwez et al., 2018), Turkish (Küçükgüçlü et al., 2011), and Hebrew (Vitman-Schorr et al., 2014). The validation studies that conducted factor analyses used exploratory methods such as PCA and EFA, with the exception of the study by Küçükgüçlü et al. (2011), which used confirmatory factor analysis (CFA). Only four studies were able to confirm the original two-factor structure (Alquwez et al., 2018; Fernández-Muñoz et al., 2023; Matarese et al., 2013; Rejeh et al., 2012); other studies identified between one (Gansukh et al., 2025) and six factors (Lambrinou et al., 2005), with the number of items reduced to as few as 18 (Ortiz-Rubio et al., 2020). The percentage of variance explained ranged from 25.69% (Fernández-Muñoz et al., 2023) to 78% (Matarese et al., 2013). Convergent validity was examined in only one study by Ortiz-Rubio et al. (2020), which demonstrated a correlation between the positive factors of the KAOP and PANAS scales as well as their respective negative factors.

To the best of our knowledge, there is no full German translation of the KAOP Scale. However, Randler et al. (2014) used eight items from the scale in their study.

#### 2.3.2 The Aging Semantic Differential (ASD)

The ASD was developed by Rosencranz and McNevin (1969) to assess attitudes and perceptual predisposition toward older adults. The scale consists of 32 pairs of bipolar adjectives with seven response levels (e.g., 1 = rich, 7 = poor), yielding a total score of 32 to 224. Lower scores represent a more positive view of older adults. Each participant needs to answer two parallel versions of the scale: the first version refers to attitudes toward younger adults (“Please indicate how you perceive younger adults”), the second to attitudes toward older adults (“Please indicate how you perceive older adults”). The German version of the ASD was translated by Stange (2003) and validated by Gluth et al. (2010) in a heterogeneous sample of younger and older adults. Intrieri et al. (1995) originally proposed a four-factor structure, which was replicated with the latent factors “instrumentality” (referring to a person’s activity and adaptability), “autonomy” (referring to a person’s autonomy and self-sufficiency), “acceptability” (referring to a person’s sociability), and “integrity” (referring to a person’s satisfaction and inner peace).

#### 2.3.3 Age stereotype and future selves

Age Stereotypes (AS) and Future Selves (FS) were assessed using the brief version of the domain-specific multidimensional approach developed by Kornadt et al. (2020). The brief version includes nine items, each relating to perceptions of life in old age and perceptions of one’s own life in old age, covering the following areas: family and partnership, friends and acquaintances, leisure activities and social or civic commitment, personality and way of living, financial situation and dealing with money-related issues, work and employment, appearance, mental and physical fitness, and health (Kornadt et al., 2020). Each item consists of bipolar evaluative statements (AS: “Older people have few friends and acquaintances”—“Older people have many friends and acquaintances,” FS: “When I am old, I will have few friends and acquaintances”—“When I am old, I will have many friends and acquaintances”). Participants rated their degree of agreement with one of the two poles on an 8-point Likert scale, ranging from 1 (less positive evaluation) to 8 (more positive evaluation), with higher scores reflecting more positive views on aging. For each domain, AS were assessed before current FS.

#### 2.3.4 The Big Five Inventory (BFI-10)

The BFI-10 is a brief German version of the original Big Five Inventory developed by John et al. (1991), which was adapted for German-speaking populations by Rammstedt (2007). The instrument is based on the five-factor model of personality and covers the following five dimensions: extraversion (items 1 and 6), agreeableness (items 2 and 7), conscientiousness (items 3 and 8), neuroticism (items 4 and 9), and openness to experience (items 5 and 10). Each dimension is measured by one item with a positive polarity and one item with a negative polarity. Items 1, 3, 4, 5, and 7 were reverse-coded prior to analysis. Responses are provided on a five-point Likert scale ranging from 1 (*strongly disagree*) to 5 (*strongly agree*). Compared to other measurement instruments, the short form with ten items is significantly shorter and has been comprehensively validated (Rammstedt et al., 2013).

### 2.4 Translation and content validity of the German version

The translation-back method (Hambleton, 2001) was used to translate the English version of the KAOP Scale into German. Specifically, two independent native German speakers translated the original English version into German. Discrepancies between the two versions were discussed within the research team to ensure conceptual equivalence and cultural appropriateness, and a reconciled German version was produced. This German version was back-translated by an English translator. The original English version and the back-translated versions were compared for consistency, relevance, and the content meaning.

An expert panel consisting of five members (two gerontologists, two psychologists, and one nurse) rated the German version based on relevance, clarity, and simplicity as 1 (*not appropriate at all*) to 4 (*completely appropriate*) (Lynn, 1986) and suggested alternative formulations. Final changes were made regarding their recommendations, and a pilot test was performed with eight participants of the general population to ensure comprehensibility and feasibility. The final German version of the KAOP (KAOP-D) is publicly available on OSF (https://osf.io/8sae5/overview).

### 2.5 Statistical analysis

The descriptive and inferential statistical analyses were conducted via IBM Statistical Package for Social Sciences (SPSS) Version 31.0. The psychometric evaluation of the KAOP included internal consistency (Cronbach’s alpha and McDonald’s omega), structural validity (principal component analysis), construct validity (employing the known-groups method), and convergent validity (correlation with ASD, AS, and FS).

#### 2.5.1 Internal consistency and test–retest reliability

Cronbach’s alpha was computed to assess internal consistency, which evaluates the reliability of scales comprising more than 10 items (Tavakol and Dennick, 2011), capturing the extent of shared variance among items. Values between 0.70 and 0.95 indicate good internal consistency, while values exceeding 0.95 may suggest item redundancy due to overlapping content (Tavakol and Dennick, 2011; Terwee et al., 2007). Besides, McDonald’s omega was calculated, as it accounts for varying unstandardized factor loadings and error variances, which can provide a potentially more robust measure of internal consistency (McDonald, 2013; Wirtz, 2026).

To assess retest reliability, data from the full sample (*N* = 419) were compared with the subsample (*n* = 211), which was assessed after a Four-week interval. The intraclass correlation coefficient (ICC) and its 95% confidence interval were calculated to measure the similarity between the two surveys based on single-rating absolute agreement using a two-way mixed-effects model. According to the 95% confidence intervals of the ICC estimates, values below 0.50 indicate poor reliability, values between 0.50 and 0.75 indicate moderate reliability, values between 0.75 and 0.90 indicate good reliability, and values above 0.90 indicate excellent reliability (Koo and Li, 2016).

#### 2.5.2 Structural validity

To examine the underlying factor structure, a principal component analysis (PCA) with varimax rotation was performed on the KAOP-D. PCA was chosen to enable direct comparison with previous validation studies, most of which have used this approach despite reporting heterogeneous factor structures. As no consensus factor structure has been established for the instrument, CFA was not considered appropriate. The PCA findings should be interpreted as exploratory, recognizing that PCA is a data reduction method rather than a latent variable approach. The criteria for conducting a PCA included a Kaiser–Meyer–Olkin (KMO) coefficient greater than 0.6 (sample size adequacy) and a significant Bartlett’s test of sphericity (suitability of the scale items for analysis) with a *p* value less than 0.05 (Kaiser and Rice, 1974; Tabachnick and Fidell, 2014) and an anti-image correlation, which is a measure of sampling adequacy, of > 0.6 for each item (Grimm et al., 2018). We applied the Guttman–Kaiser eigenvalue-greater-than-one rule to reconstruct a lower-dimensional dataset that still included adequate sources of variance and confirmed the results using a scree plot (Tabachnick and Fidell, 2014). The interpretability and theoretical coherence of the factor solution were also considered when determining the final component structure.

#### 2.5.3 Construct validity

Construct validity was assessed by the known-groups method. The Shapiro–Wilk test was used to assess data distribution for normality. Depending on the data distribution, between-group differences were analyzed using an independent-samples *t-test* for normally distributed data or a Wilcoxon–Mann–Whitney test for non-normally distributed data. Statistical significance was set at *p* < 0.05. Groups were defined based on variables for which differences in attitudes toward older adults have been reported in previous research. Accordingly, known-groups analyses were conducted using gender, education, and attitudes toward older adults as grouping variables. Regarding age differences in attitudes toward older people, some studies reporting more positive attitudes among older adults (e.g., Guo, 1999), while others have found no differences or more positive attitudes among younger adults (e.g., Rothbaum, 1983). Overall, however, the balance of evidence suggests that older adults tend to hold more positive attitudes toward aging than younger adults. Therefore, we formulated the following hypothesis for this study: (1) Older participants were expected to report more positive attitudes toward older adults than younger participants; (2) female participants were expected to report more positive attitudes toward older adults than male participants; (3) participants with a higher educational level were expected to report more positive attitudes toward older adults than those with a lower educational level; (4) participants with a positive attitude toward older age will score higher in the KAOP Scales than people with a negative attitude.

#### 2.5.4 Convergent validity

Convergent validity was assessed via correlations, whereby tests involving identical or similar constructs (ASD, AS, and FS) were expected to show significant correlations (Chin and Yao, 2014).

### 2.6 Ethical

This study was approved by the Ethics Committee of the Faculty of Behavioral and Empirical Cultural Sciences, Heidelberg University (AZ Teich 2025 2/1). All procedures involved in this work conformed to the ethical standards of the Declaration of Helsinki, as applicable to national and institutional human experimentation committees. Prior to the survey, we obtained written informed consent from each participant. The participants were informed that the research was voluntary, confidential, and for academic purposes only. After merging the data from different time points to ensure anonymity, the research team removed the email addresses from the server. Data processing complied with the General Data Protection Regulation (GDPR).

## 3 Results

### 3.1 Participant characteristics

A total of *N* = 419 participated in the study, of whom *n* = 211 completed the questionnaire a second time after 4 weeks. Table 1 shows the sociodemographic data for both the total sample and the subgroup as well as the differences (*t-test* or χ^2^). The median age of the total sample was 56 years (range: 18–90 years) and 60 years for the subgroup, with the majority of participants being female (68.3 and 68.7%, respectively). The most common level of education attained was 12 to 13 years of education, followed by a Master’s degree or diploma, accounting for 25.5 and 21.5% of the total sample and 23.7 and 23.7% of the subgroup. Most participants reported good to very good subjective health status (66.6%) with no chronic illness (70.0%) and 45.3% live with their partner.

**Table 1:** Participant characteristics of the total sample and the subgroup.

Characteristics	Full sample (*N* = 419)		Subgroup (*n* = 211)	
	*n*	%	*n*	%
Age				
Mean	51.15		55.38	
Median	56.00		60.00	
SD	19.55		18.35	
Gender				
Male	132	31.5	66	31.3
Female	286	68.3	145	68.7
Diverse	1	0.2	0	0.0
Education				
9 years or less	7	1.6	2	0.9
10 years	44	10.7	22	10.4
12–13 years	107	25.5	50	23.7
Vocational training	23	5.5	12	5.7
Bachelor	40	9.5	19	9.0
Master/Diploma	90	21.5	50	23.7
PhD	57	13.6	25	11.8
Other	51	12.2	31	14.7
Occupation				
School student	2	0.5	1	0.5
Student	75	17.9	23	10.9
Unemployed	5	1.2	3	1.4
Retiree	107	25.5	67	31.8
Employee	149	35.6	78	37.0
Civil servant	27	6.4	10	4.7
Self-employed	19	4.5	10	4.7
Housewife/househusband	8	1.9	5	2.4
Other	27	6.4	14	6.6
Marital status				
Divorced	28	6.7	15	7.1
In partnership or married	252	60.1	135	64.0
Single	118	28.2	47	22.3
Widowed or deceased partner	21	5.0	14	6.6
Do you have children?				
Yes	236	56.3	125	59.2
No	185	43.7	86	40.8
Country				
Germany	401	95.7	201	95.3
Austria	5	1.2	3	1.4
Switzerland	1	0.2	0	0.0
Other	12	2.9	7	3.3
Type of housing				
Alone	95	22.7	40	19.9
With partner/husband/wife	189	45.1	110	52.1
With child(ren)	14	3.3	8	3.8
With partner and child(ren)	62	14.8	26	12.3
Other	59	14.1	27	12.8
Health status				
Poor	3	0.7	2	0.9
Less good	31	7.4	11	5.2
Satisfied	107	25.5	45	21.3
Good	193	46.1	111	52.6
Very good	85	20.3	42	19.9
Do you have a chronic illness?				
Yes	124	30.3	62	29.4
No	289	69.7	149	70.6
Life satisfaction^1^				
Mean	7.62		7.82	
Median	8.00		8.00	
SD	1.76		1.63	
Do you have difficulty performing activities of daily living (shopping, bathing, getting dressed, etc.)?				
No	372	88.8	189	89.6
Slight	35	8.4	19	9.0
Moderate	7	1.7	1	0.5
Significant	3	0.7	0	0.0
Very significant	2	0.5	2	0.9
Pension^2^				
Yes	296	70.6	167	79.1
No	100	23.9	36	17.1
I do not know	23	5.5	8	3.8

Personality	Mean	SD	Mean	SD
Extraversion	3.37	0.98	3.41	1.00
Agreeableness	3.41	0.74	3.47	0.72
Conscientiousness	3.88	0.77	3.91	0.74
Neuroticism	2.79	0.99	2.75	0.99
Openness to experience	3.86	0.89	3.91	0.91

^1^Life satisfaction was assessed by a single item with a 10-point response scale from 1 = very dissatisfied to 10 = very satisfied. ^2^Do you have savings or a financial plan for retirement?

Additionally, the experience with older adults was determined (Table 2), showing no significant gender differences. Although only 22.4% of the participants still have grandparents, 72.3% live in a neighborhood with older adults. When asked about formative influences on their image of old age, 34.4% reported that their views were shaped by individuals other than their grandparents, whereas 25.0% attributed this influence primarily to their grandmother. In contrast, only 7.5% reported that their grandfather had influenced their image of old age.

**Table 2:** Experience with older adults.

Item (*N*, %)	Total (*N* = 419)	Women (*n* = 286)	Men (*n* = 132)	*p*
Do older adults live in the neighborhood?				
Yes, some	303 (72.3)	211 (73.8)	92 (69.7)	0.650
Yes, but only few	100 (23.9)	64 (22.4)	35 (26.5)	
No	16 (3.8)	11 (3.8)	5 (3.8)	
Do you still have grandparents?				
Yes	94 (22.4)	65 (22.7)	28 (21.2)	0.851
No	325 (77.6)	221 (77.3)	104 (78.8)	0.729
Where do our grandparents live or where did they live?				
In the same house	58 (13.8)	37 (12.9)	21 (15.9)	0.414
In the neighborhood	19 (4.5)	13 (4.5)	6 (4.5)	1.000
In the same town	73 (17.4)	49 (17.1)	24 (18.2)	0.793
In a town nearby	103 (24.6)	78 (27.3)	24 (18.2)	**0.044**
In a nursing home nearby	29 (6.9)	20 (7.0)	9 (6.8)	0.948
In a nursing home far away	16 (3.8)	9 (3.1)	7 (5.3)	0.286
Far away	183 (43.7)	126 (44.1)	57 (43.2)	0.867
Unfortunately, I never got to know my grandparents or had any contact with them.	32 (7.7)	18 (6.3)	14 (10.6)	0.128
How often do you see the grandparents you see most often on average, or have seen them?				
Daily	42 (0.2)	27 (9.7)	15 (11.7)	0.656
Several times a week	52 (12.7)	35 (12.5)	17 (13.3)	
Once a week	36 (8.8)	27 (9.7)	9 (7.0)	
Several times a month	43 (10.5)	31 (11.1)	11 (8.6)	
Once a month	30 (7.4)	24 (8.6)	6 (4.7)	
Several times a year	110 (27.0)	76 (27.2)	34 (26.6)	
Once a year	32 (7.8)	21 (7.5)	11 (8.6)	
Less than once a year	28 (6.9)	18 (6.5)	10 (7.8)	
I have/had no contact with my grandparents	34 (8.3)	19 (6.8)	15 (11.7)	
Who has influenced your view of old age the most?				
Grandmother	104 (25.0)	75 (26.4)	29 (22.1)	0.546
Grandfather	31 (7.5)	20 (7.0)	11 (8.4)	
Other person	143 (34.4)	101 (35.6)	42 (32.1)	
No one	55 (13.2)	33 (11.6)	22 (16.8)	
I do not know	83 (20.0)	55 (19.4)	27 (20.6)	

Bold values indicate statistical significance (*p* < 0.05).

Finally, we asked the participants about their perception of older adults (Table 3). While only a few people cited physical characteristics such as gray hair or wrinkles as signs of old age, 29.1% of participants responded that someone aged 70 or older can be considered old, and 22.4% believed that people aged 75 or older are considered old.

**Table 3:** Perceptions of older adults.

Item (*N*, %)	Total (*N* = 419)	Women^1^ (*n* = 286)	Men (*n* = 132)	*p*
At what age does someone count as “old”?				
When you start retirement	84 (20.1)	55 (19.2)	29 (22.0)	0.516
When someone has gray hair	3 (0.7)	2 (0.7)	1 (0.8)	0.948
When someone does not know how to have fun anymore	24 (5.7)	13 (4.5)	11 (8.3)	0.122
When someone has wrinkles	2 (0.5)	2 (0.7)	0 (0.0)	0.336
From age of 40	5 (1.2)	2 (0.7)	3 (2.3)	0.169
From age of 50	8 (1.9)	7 (2.4)	1 (0.8)	0.241
From age of 60	42 (10.0)	25 (8.7)	17 (12.9)	0.191
From age of 65	63 (15.0)	45 (15.7)	18 (13.6)	0.577
From age of 70	122 (29.1)	83 (29.0)	39 (29.5)	0.913
From age of 75	94 (22.4)	74 (25.9)	20 (15.2)	**0.015**
From age of 80	72 (17.2)	54 (18.9)	18 (13.6)	0.187
From age of 85	18 (4.3)	14 (4.9)	4 (3.0)	0.383
From age of 90	10 (2.4)	8 (2.8)	2 (1.5)	0.425
Other	24 (5.7)	13 (4.5)	10 (7.6)	0.207
Can you imagine working in a nursing home/elderly care/geriatrics later on?				
Yes	105 (25.1)	79 (27.6)	26 (19.7)	0.082
No	198 (47.3)	128 (44.8)	69 (52.3)	0.152
Maybe	124 (29.6)	84 (29.4)	40 (30.3)	0.846
What is your attitude toward older adults?				
Positive	147 (35.1)	97 (33.9)	50 (37.9)	0.704
Neutral	223 (53.2)	154 (53.8)	68 (51.5)	
Negative	49 (11.7)	35 (12.2)	14 (10.6)	
What topics concern you most in relation to age?				
Health	362 (86.4)	245 (85.7)	117 (88.6)	0.407
Loneliness	189 (45.1)	139 (48.6)	49 (37.1)	**0.028**
Financial insecurity	160 (38.2)	119 (41.6)	40 (30.3)	**0.027**
Dependence on care	256 (61.1)	193 (67.5)	63 (47.7)	**<0.001**
Abuse/discrimination	37 (8.8)	31 (10.8)	6 (4.5)	**0.035**
Other	31 (7.4)	22 (7.7)	9 (6.8)	0.751
Have you worked with older adults in your profession or job?				
Yes	289 (69.0)	195 (68.2)	93 (70.5)	0.641
No	130 (31.0)	91 (31.8)	39 (29.5)	
Have you ever visited a nursing home or geriatric clinic?				
Yes	367 (87.6)	253 (88.5)	113 (85.6)	0.411
No	52 (12.4)	33 (11.5)	19 (14.4)	
Compared to your current age, how old do you feel?				
Much younger	84 (20.0)	57 (19.9)	27 (20.5)	0.973
Slightly younger	207 (49.4)	140 (49.0)	67 (50.8)	
Roughly my age	101 (24.1)	70 (24.5)	31 (23.5)	
Slightly older	22 (5.3)	15 (5.2)	6 (4.5)	
Much older	5 (1.2)	4 (1.4)	1 (0.8)	

^1^As there was only one participant diverse, the person was not included in the analysis.Bold values indicate statistical significance (*p* < 0.05).

Almost half of the participants cannot imagine working in a nursing home or geriatric ward. Most participants had a neutral attitude toward age, and only 11.7% reported a negative attitude. The participants were most concerned about their health in old age (86.4%), followed by dependence on the care of others (61.1%) and loneliness (45.1%). Notably, there were gender-specific differences, particularly in the areas of loneliness, dependence on others for care, and experiencing abuse or discrimination. These issues were reported significantly more often by women.

### 3.2 Descriptive statistics

Table 4 displays the psychometric properties for all included scales. The total score of the sample was 142.53 (SD = 18.34), indicating moderately positive attitudes toward older adults (possible range: 34–204). The total scores for the negative and positive items were 75.42 (SD = 10.67) and 67.11 (SD = 10.16), respectively.

**Table 4:** Psychometric properties of all scales.

Scale (number of items)	Mean (SD) [range]	Cronbach’s alpha			McDonald’s omega	Mean item-total correlation	Mean inter-item correlation
	Total sample	Total sample	N^1^ (*n* = 49)	P^2^ (*n* = 147)	Total sample	Total sample	Total sample
KAOP-D-34 (34)	4.192 (0.688) [1,6]	0.870	0.908	0.870	0.863	0.386	0.171
KAOP-N-D (17)	4.437 (0.644) [1,6]	0.826	0.860	0.822	0.826	0.436	0.231
KAOP-P-D (17)	3.947 (0.658) [1,6]	0.784	0.789	0.833	0.780	0.375	0.226
KAOP-D-32 (32)	**4.207 (0.706) [1,6]**	**0.874**	**0.918**	**0.870**	**0.867**	**0.403**	**0.186**
ASD (young) (32)	3.605 (0.421) [1,7]	0.934	0.919	0.943	0.972	0.533	0.303
ASD (old) (32)	3.605 (0.519) [1,7]	0.934	0.940	0.945	0.950	0.535	0.305

ASD: Aging Semantic Differential; KAOP-D: German version of the Kogan’s Attitudes toward Old People Scale; KAOP-N-D: negatively worded items; KAOP-P-D: positively worded items; N: self-assessed negative attitude toward older adults; P: self-assessed positive attitude toward older adults. ^1,2^Participants who answered that their attitude toward older adults is neutral were not included (*n* = 223). Bold values indicate the psychometric properties of the final 32-item version retained after item reduction.

### 3.3 Validation of the KAOP Scale

#### 3.3.1 Preliminary item statistics

Corrected item-total correlations ranged from 0.13 to 0.60 (Table 5). Most items showed moderate to strong associations with the total score, indicating satisfactory item discrimination. However, three items displayed notably low corrected item-total correlations: item 7 (*r* = 0.13), item 8 (*r* = 0.16), and item 24 (*r* = 0.17), whereas items 2, 11, 12, 13, and 18 showed correlations between 0.20 and 0.30. These coefficients fall well below the commonly accepted threshold of 0.30, suggesting that the items contribute little to the underlying construct and may need to be revised or removed from the final scale.

**Table 5:** Item-level descriptive statistics, corrected item-total correlations, and factor loadings for the KAOP Scale.

Item	Mean	SD	Item-total correlations	α if item deleted	Factor 1	Factor 2	Communality (h^2^)
01	4.98	1.225	0.425	0.865	0.576		0.332
02	4.83	1.273	**0.288**	0.869	0.361		**0.133**
03	4.26	1.273	0.344	0.867	0.481		0.232
04	3.54	1.426	0.401	0.866	0.361	0.290	0.215
05	3.65	1.264	0.488	0.864	0.512	0.241	0.320
06	3.67	1.193	0.400	0.866	0.371	0.287	0.220
07	3.90	1.260	**0.128**	**0.872**	**0.160**		**0.026**
08	4.00	1.290	**0.156**	**0.872**		**0.173**	**0.034**
09	5.19	0.972	0.402	0.866	0.575		0.330
10	5.08	1.069	0.331	0.868	0.390		0.159
11	3.29	1.496	**0.228**	**0.871**		0.414	**0.172**
12	3.34	1.285	0.303	0.868		0.574	0.329
13	3.53	1.535	**0.234**	**0.871**		0.484	**0.234**
14	3.00	1.306	0.312	0.868		0.633	0.401
15	5.24	1.108	0.480	0.865	0.607		0.376
16	4.27	1.192	0.494	0.864	0.298	0.547	0.388
17	4.37	1.221	0.470	0.864	0.467	0.264	0.288
18	4.52	1.127	0.201	0.870		0.372	**0.139**
19	4.32	1.228	0.596	0.862	0.567	0.371	0.459
20	3.52	1.242	0.450	0.865	0.285	0.518	0.349
21	4.38	1.329	0.430	0.865	0.517	0.121	0.282
22	4.81	1.282	0.552	0.862	0.552	0.276	0.381
23	5.31	1.032	0.373	0.867	0.525		**0.276**
24	3.08	1.385	**0.167**	**0.872**	−0.120	0.510	**0.275**
25	4.66	1.210	0.379	0.867	0.570		0.328
26	4.15	1.347	0.397	0.866	0.380	0.245	0.204
27	5.14	0.967	0.395	0.866	0.598		0.361
28	4.48	1.050	0.379	0.867	0.365	0.211	**0.177**
29	4.83	1.018	0.566	0.863	0.638	0.214	0.453
30	3.87	1.040	0.452	0.865	0.242	0.542	0.352
31	3.73	1.312	0.551	0.862	0.540	0.340	0.407
32	2.99	1.185	0.469	0.865	0.239	0.608	0.427
33	4.66	1.131	0.509	0.864	0.629	0.110	0.408
34	3.97	1.609	0.366	0.867	0.366	0.206	**0.176**
Eigenvalue					5.985	3.660	
Cronbach’s α					0.864	0.711	
Total variance explained					**17.60%**	**10.77%**	**Σ 28.37%**

Bold values indicate items considered for removal during item reduction: low corrected item-total correlations (<0.30); increase in Cronbach’s α when deleted; low factor loadings (<0.20); low communalities (<0.30), as well as the variance explained by the two extracted factors and their cumulative contribution to the total variance.

#### 3.3.2 Validity

##### 3.3.2.1 Content validity

The scale’s content validity was evaluated using the Content Validity Index (CVI). Item-level CVI (I-CVI) indicated that all items were rated as either “appropriate” or “very appropriate,” by the five experts, with the exception of items 7 and 8, which were deemed inappropriate for the German context. The scale-level CVI (S-CVI), calculated using the averaging method, was 0.94, indicating excellent overall content validity. Interrater agreement was assessed using Kendall’s coefficient of concordance, yielding a statistically significant level of agreement among experts (Kendall’s W = 0.56, *p* < 0.001).

##### 3.3.2.2 Structural validity

Given the absence of a validated German version of the KAOP Scale and the heterogeneity of factor structures reported across international adaptations, a PCA was conducted on the full sample (*N* = 419) to examine the underlying factor structure. The criteria for PCA were met with KMO of 0.822 and a significant Bartlett’s test of sphericity χ^2^ (561) = 4,439.426, *p* ≤ 0.001. An initial PCA extracted 11 factors with eigenvalues greater than 1, explaining 64.17% of the total variance. However, the scree plot showed a clear elbow after the second factor, indicating that a two-factor solution was more appropriate. Therefore, the PCA with two factors was conducted (Table 5).

Item-total correlations were comparatively low for items 2, 7, 8, 11, 13, and 24. Removal of these items would have resulted in a slight increase in Cronbach’s alpha, with the exception of item 2. Communalities (h^2^) were low for items 2, 7, 8, 11, 18, 28, and 34, indicating limited representation within the extracted factor structure. Measures of sampling adequacy (MSA) values exceeded the threshold of 0.6 for all items except items 7, 8, and 11 (0.473, 0.504, 0.561, respectively; see Supplementary material 2).

Item reduction was guided by the following predefined criteria: (1) a factor loading of at least 0.30 on one factor; (2) retention of paired items (positive–negative wording); (3) an MSA value ≥ 0.60; and (4) considerations of content validity. Based on these criteria, items 7 and 8 were removed. The PCA was subsequently repeated with the remaining 32 items (Table 6).

**Table 6:** PCA of the KAOP Scale with 32 items.

Item	Item content	Factor 1	Factor 2	*r*
1	It would probably be better if most old people lived in residential units with people of their own age.*	0.576		0.427
2	It would probably be better if most old people lived in residential units that also housed younger people.	0.368		0.284
3	There is something different about most old people: it’s hard to figure out what makes them tick.*	0.480		0.346
4	Most old people are really no different from anybody else: they are as easy to understand as younger people.	0.371		0.403
5	Most old people get set in their ways and are unable to change.*	0.510		0.483
6	Most old people are capable of new adjustments when the situation demands it.	0.383		0.404
9	Most old people tend to let their homes become shabby and unattractive.*	0.575		0.406
10	Most old people can generally be counted on to maintain a clean, attractive home.	0.402		0.326
11	It is foolish to claim that wisdom comes with old age.*		0.424	0.230
12	People grow wiser with the coming of old age.		0.573	0.308
13	Old people have too much power in business and politics.*		0.497	0.236
14	Old people should have more power in business and politics.		0.634	0.304
15	Most old people make one feel ill at ease.*	0.608		0.476
16	Most old people are very relaxing to be with.	0.317	0.528	0.495
17	Most old people bore others by their insistence on talking about the “good old days.”*	0.468		0.464
18	One of the most interesting and entertaining qualities of most old people is their accounts of their past experiences.		0.365	0.197
19	Most old people spend too much time prying into the affairs of others and giving unsought advice.*	0.573	0.366	0.602
20	Most old people tend to keep to themselves and give advice only when asked.	0.302	0.512	0.466
21	If old people expect to be liked, their first step is to try to get rid of their irritating faults.	0.515		0.435
22	When you think about it, old people have the same faults as anybody else.	0.563		0.545
23	In order to maintain a nice residential neighborhood, it would be best if too many old people did not live in it.	0.527		0.377
24	You can count on finding a nice residential neighborhood when there is a sizeable number of old people living in it.		0.508	0.164
25	There are a few exceptions, but in general most old people are pretty much alike.	0.568		0.381
26	It is evident that most old people are very different from one another.	0.390		0.405
27	Most old people should be more concerned with their personal appearance; they are too untidy.*	0.597		0.400
28	Most old people seem to be quite clean and neat in their personal appearance.	0.373		0.380
29	Most old people are irritable, grouchy, and unpleasant.*	0.640		0.573
30	Most old people are cheerful, agreeable, and good humored.		0.529	0.460
31	Most old people are constantly complaining about the behavior of the younger generation.*	0.544	0.338	0.559
32	One seldom hears old people complaining about the behavior of the younger generation.		0.610	0.475
33	Most old people make excessive demands for love and reassurance.	0.630		0.515
34	Most old people need no more love and reassurance than anyone else.	0.376		0.363
Eigenvalue		6.063	3.536	
Cronbach’s α		0.869	0.726	
Total variance explained		18.95	11.05	**Σ 30.00**

*r* = corrected item-total correlation; *reverse-coded item; odd-numbered items are negative items, even items are positive items. Bold value indicates the variance explained by the two extracted factors and their cumulative contribution to the total variance.

The retained two-component solution accounted for 30.0% of the total variance. Factor 1 reflects socially distancing and exclusionary attitudes toward older adults, including perceptions of rigidity, interpersonal discomfort, and normative deviance (e.g., “Most old people are irritable, grouchy, and unpleasant”). Factor loadings ranged from 0.368 to 0.640. Factor 2 reflects attributions of social value, competence, and interpersonal warmth, including perceptions of wisdom, cheerfulness, and societal contribution (e.g., “People grow wiser with the coming of old age”). Factor loadings ranged from 0.365 to 0.634.

A small number of items exhibited secondary loadings above 0.30. However, none of these exceeded the 0.40 threshold for salient cross-loadings, and in all cases the primary loading was substantially higher, with differences exceeding 0.20.

##### 3.3.2.3 Construct validity

Table 7 presents the results of the known-groups analyses applied to gender, age, education, and attitude toward older adults using the Wilcoxon–Mann–Whitney test (WMW) (Mann and Whitney, 1947), since the distribution of scale scores deviated from normality, as shown by the Shapiro–Wilk test. The findings indicate that all versions of the KAOP-D successfully differentiated between participants based on these four variables. For the 32-item version (KAOP-D-32), factor 2 could not differentiate between participants based on gender and education, while KAOP-D-P did not differentiate between participants based on education.

**Table 7:** Known-groups analysis for all versions of the KAOP-D Scale.

Scale	Gender		Age		Education		Attitude		*p*
	Female (286)	Male (132)	Young^1^ (98)	Older^2^ (235)	Non-acad. (183)	Acad. (236)	Pos. (147)	Neg. (49)	
	Mean rank								
KAOP-D-34	220.04	186.66	114.71	188.81	193.46	222.83	107.50	71.49	Gender* Αge** Education* Attitude**
KAOP-D-N	219.42	188.00	124.51	184.72	189.55	225.86	108.75	67.76	Gender* Αge** Education* Attitude**
KAOP-D-P	217.40	192.38	116.13	188.21	199.62	218.05	105.52	77.45	Gender* Αge** Attitude*
KAOP-D-32	219.66	187.48	113.40	189.35	192.35	223.69	107.29	72.12	Gender* Αge** Education* Attitude**
KAOP-D F1	220.10	186.53	118.48	187.23	186.09	228.54	108.04	69.88	Gender* Αge** Education** Attitude**
KAOP-D F2	214.45	198.78	127.31	183.55	209.05	210.74	103.64	83.09	Αge** Attitude*

KAOP-D: German version of the Kogan’s Attitudes toward Older People Scale; ^1^Young: 18–30 years; ^2^Older: 50+ years; WMW tests were used for all analyses; *p < 0.05; **p < 0.001.

##### 3.3.2.4 Convergent validity

Table 8 provides an overview of the correlations between the different versions of KAOP-D and the ASD, personality, age, health, life satisfaction, pension, and subjective age. As almost all scales except the factor 2 of KAOP-D-32 and ASD young were not normally distributed (significant Shapiro–Wilk test), Spearman’s rank-order correlations were calculated.

**Table 8:** Heatmap for the correlations between the scales and other variables.

Scale/Item	1	2	3	4	5	6	7	8	9	10	11	12	13	14	15	16	17
1. KAOP-D-34																	
2. KAOP-D-32	**0.99** ^**^																
3. KAOP-D F1	**0.92** ^**^	**0.93** ^**^															
4. KAOP-D F2	**0.67** ^**^	**0.68** ^**^	**0.38** ^**^														
5. KAOP-D-N	**0.87** ^**^	**0.86** ^**^	**0.88** ^**^	**0.43** ^**^													
6. KAOP-D-P	**0.87** ^**^	**0.87** ^**^	**0.73** ^**^	**0.76** ^**^	**0.54** ^**^												
7. ASD Young	**−0.13** ^**^	**−0.12** ^*^	**−0.11** ^*^	**−0.10** ^*^	**−0.12** ^*^	**−0.13** ^**^											
8. ASD Old	**−0.39** ^**^	**−0.40** ^**^	**−0.32** ^**^	**−0.37** ^**^	**−0.32** ^**^	**−0.38** ^**^	**0.29** ^**^										
9. Extraversion	**0.13** ^**^	**0.14** ^**^	**0.12** ^*^	**0.11** ^*^	0.09	**0.15** ^**^	−0.04	−0.07									
10. Agreeableness	**0.13** ^**^	**0.14** ^**^	**0.13** ^**^	0.09	**0.12** ^*^	**0.12** ^*^	**−0.18** ^**^	−0.06	**0.10** ^*^								
11. Conscientiousness	**0.15** ^**^	**0.15** ^**^	**0.14** ^**^	**0.13** ^**^	**0.13** ^**^	**0.16** ^**^	−0.04	−0.04	**0.17** ^**^	0.09							
12. Neuroticism	**−0.11** ^*^	**−0.11** ^*^	−0.10	−0.08	−0.09	**−0.12** ^*^	0.05	0.00	**−0.23** ^**^	**−0.17** ^**^	**−0.21** ^**^						
13. Openness	**0.15** ^**^	**0.14** ^**^	**0.16** ^**^	0.03	**0.15** ^**^	**0.10** ^*^	−0.05	**−0.12** ^*^	**0.10** ^*^	−0.00	0.09	−0.00					
14. Age	**0.29** ^**^	**0.30** ^**^	**0.25** ^**^	**0.26** ^**^	**0.20** ^**^	**0.33** ^**^	−0.06	0.00	0.05	−0.02	**0.23** ^**^	**−0.32** ^**^	0.01				
15. Health^1^	**0.11** ^*^	**0.12** ^*^	**0.14** ^**^	0.03	**0.12** ^*^	0.09	−0.07	−0.02	**0.16** ^**^	**0.16** ^**^	**0.23** ^**^	**−0.28** ^**^	0.07	0.03			
16. Life satisfaction^2^	**0.22** ^**^	**0.21** ^**^	**0.23** ^**^	0.08	**0.19** ^**^	**0.20** ^**^	**−0.17** ^**^	**−0.09**	**0.22** ^**^	**0.16** ^**^	**0.25** ^**^	**−0.37** ^**^	**0.11** ^*^	**0.27** ^**^	**0.55** ^**^		
17. Pension^3^	**−0.18** ^**^	**−0.19** ^**^	**−0.19** ^**^	−0.09	**−0.17** ^**^	**−0.18** ^**^	0.07	0.05	−0.08	0.05	**−0.20** ^**^	**0.15** ^**^	0.02	**−0.43** ^**^	**−0.12** ^*^	**−0.23** ^**^	
18. Subjective age^4^	**−0.12** ^*^	**−0.12** ^*^	**−0.11** ^*^	−0.08	**−0.11** ^*^	**−0.11** ^*^	−0.05	0.06	−0.04	−0.08	−0.06	**0.20** ^**^	−0.05	**−0.32** ^**^	**−0.23** ^**^	**−0.21** ^**^	**0.24** ^**^

KAOP-D: German version of the Kogan’s Attitudes toward Older People Scale; ASD: Aging Semantic Differential; Health (1 = poor to 5 = very good); ^2^Life satisfaction (1 = very dissatisfied to 10 = very satisfied); ^3^Do you have savings or a financial plan for retirement? (1 = yes, 2 = no); ^4^Subjective age (1 = much younger to 5 = much older); *significant at level *p* < 0.05; **significant at level *p* < 0.001. The color saturation has been adjusted for better visualization and is not proportional to the numerical value. Bold values indicate statistical significance (*p* < 0.05).

The different versions of the KAOP-D scales exhibited strong positive correlations with one another. Since low ASD values indicate a positive attitude, the correlations with ASD were significantly negative and stronger regarding older adults than younger people. The KAOP-D-34 and KAOP-D-32 total scores significantly correlated with extraversion, agreeableness, conscientiousness, and openness to experience. However, neuroticism negatively correlated with these scales. Age, health, and life satisfaction were positively associated with KAOP-D scores, whereas subjective age was negatively associated with KAOP-D scores.

The KAOP-D-34 and KAOP-D-32 total scores are positively and significantly correlated with most AS domains (family, friendship, leisure, personality, work, appearance, fitness, and health), with correlations ranging from 0.13 to 0.22 (*p* < 0.05/0.01). In contrast, the finance item shows a negligible, non-significant association. Their correlations with the FS domains, however, are moderate to strong (*r* ≈ 0.15–0.36, *p* < 0.05/0.01) for most domains, particularly personality, work, appearance, and fitness. Again, the finance domain yields an essentially zero, non-significant correlation (Table 9).

**Table 9:** Heatmap for the correlations between the KAOP-D scales and the dimensions of AS and FS.

Scale/Domain		KAOP-D-34	KAOP-D-32
AS	Family	0.21^**^	0.21^**^
	Friendship	0.13^*^	0.13^**^
	Leisure	0.19^**^	0.19^**^
	Personality	0.22^**^	0.22^**^
	Finance	0.01	0.02
	Work	0.19^**^	0.20^**^
	Appearance	0.21^**^	0.22^**^
	Fitness	0.20^**^	0.19^**^
	Health	0.18^**^	0.17^**^
FS	Family	0.23^**^	0.22^**^
	Friendship	0.15^**^	0.16^**^
	Leisure	0.26^**^	0.28^**^
	Personality	0.36^**^	0.36^**^
	Finance	−0.04	−0.03
	Work	0.34^**^	0.33^**^
	Appearance	0.34^**^	0.35^**^
	Fitness	0.36^**^	0.36^**^
	Health	0.25^**^	0.25^**^

AS, Age Stereotypes; FS, Future Selves.

#### 3.3.3 Reliability

##### 3.3.3.1 Internal consistency

The internal consistency of the KAOP-D-34 scale was good (Cronbach’s *α* = 0.870, McDonald’s *ω* = 0.863), with its positive and negative subscales showing acceptable to good reliability. The KAOP-D-32 scale demonstrated improved internal consistency (α = 0.874), with the two factors demonstrating α values of 0.869 and 0.726, respectively. The reliability for the other scales was good to excellent (α = 0.752–0.934, McDonald’s ω = 0.751–0.971; see Table 4).

##### 3.3.3.2 Test–retest reliability

Table 10 includes the ICC for KAOP-D as well as the sub-scales and the improved scale with their respective 95%-CI. Overall, all results indicate stable scores over time, supporting the temporal reliability of the KAOP-D and its derived subscales.

**Table 10:** Intraclass correlation coefficient for the KAOP-D scale.

Scale (number of items)	Intraclass correlation coefficient	95% – Confidence Interval	
		Lower bound	Upper bound
KAOP-D-34 (34)	0.846	0.802	0.880
KAOP-D-N (17)	0.792	0.736	0.838
KAOP-D-p (17)	0.752	0.670	0.814
KAOP-D-32 (32)	0.857	0.816	0.889
KAOP-D-32 Factor 1 (22)	0.831	0.784	0.869
KAOP-D-32 Factor 2 (10)	0.769	0.698	0.824

## 4 Discussion

The objective of this study was to translate, adapt, and validate the KAOP-D-34 for the German population. The scale-level CVI indicated excellent overall content validity, except for items 7 and 8, which were excluded from the scale. This yielded the final KAOP-D-32. PCA revealed two distinct factors. All versions of the scale could distinguish between gender, age groups, education, and attitudes toward older people. However, Factor 2 could not distinguish between participants based on gender or education. Additionally, the subscale comprising positive statements (KAOP-D-P) could not distinguish between participants based on education.

All variants demonstrated good internal consistency, with only the KAOP-D-P and Factor 2 of the KAOP-D-32 showing slightly lower, yet still acceptable, values. All scales showed good test–retest reliability, supporting the temporal reliability of the KAOP-D and its derived subscales.

Although there are many validation studies in various languages, the analyses are not always comparable. First of all, although most studies used a rating of 1 to 6 as we did in our study, yielding a total score range of 34–204 (Alquwez et al., 2018; Erdemir et al., 2011; Fernández-Muñoz et al., 2023; Lambrinou et al., 2005; Ortiz-Rubio et al., 2020; Vitman-Schorr et al., 2014; Doherty et al., 2011; Wanko Keutchafo and Kerr, 2024), others applied a rating of 1 to 7. In these cases, a score of 4 was assigned to items that received no response, yielding a total range of 34–238 (Küçükgüçlü et al., 2011; Rejeh et al., 2012; Runkawatt et al., 2016; Ogiwara et al., 2007; Matarese et al., 2013; Yen et al., 2009; Iwasaki and Jones, 2008). Additionally, some studies utilized a score of 1 to 5 with a total range of 34–170 (Söderhamn et al., 2000), with only 32 items, yielding a total range of 32–160 (Abudu-Birresborn et al., 2023), or did not report the scoring used at all (Da Perez et al., 2024; Gansukh et al., 2025).

Content validity in the present study was high (CVI = 0.94), indicating strong expert agreement on the relevance and representativeness of the KAOP items. This finding is consistent with previous cross-cultural validation studies of the instrument, which fall within a comparable range (Erdemir et al., 2011; Küçükgüçlü et al., 2011; Rejeh et al., 2012; Runkawatt et al., 2016). Slightly lower values were reported in the Italian (Matarese et al., 2013) and Mongolian validation studies (Gansukh et al., 2025).

Due to the heterogeneity of reported factor structures across international adaptations, a PCA was conducted in the present study, revealing a two-factor solution. Unlike most studies with a two-factor solution, the two factors did not consist of only negative or only positive items. Factor 1 reflects attitudes toward older adults that involve social distancing and exclusion, including perceptions of rigidity, interpersonal discomfort, and normative deviance. Factor 2 reflects attributions of social value, competence, and interpersonal warmth, including perceptions of wisdom, cheerfulness, and societal contribution. Despite the general support for the underlying construct, factor-analytic findings across KAOP validation studies show some methodological variability. Most studies used exploratory approaches such as PCA or EFA, occasionally followed by CFA, and retained the original 34-item structure. A clear two-factor solution with Factor 1 (*Prejudice*) consisting of 17 negative items and Factor 2 (Appreciation) consisting of 17 positive items could be seen in the Taiwanese version (Yen et al., 2009), the Arabic version (Alquwez et al., 2018), the Iranian validation (Rejeh et al., 2012), as well as in the Spanish (Fernández-Muñoz et al., 2023) and the Italian versions (Matarese et al., 2013). Nonetheless, the last two mentioned displayed some weak factor loadings. The two-factor structure could be confirmed with CFA in the Turkish version (Küçükgüçlü et al., 2011).

However, several adaptations reported deviations from the original structure. The Spanish validation study by Ortiz-Rubio et al. (2020) and the Cameroonian validation study by Wanko Keutchafo and Kerr (2024) identified a two-factor solution, which was reduced to 18 and 22 items, respectively, following factor analysis. Although the Turkish validation study by Erdemir et al. (2011) also reported a two-factor solution, most of the items loaded onto both factors with little difference in loading.

A three-factor solution (*Prejudice, Appreciation,* and *Resemblance*) was reported by Söderhamn et al. (2000) in the Swedish version, with 4 items showing loadings lower than 0.3. Also, the Japanese version by Ogiwara et al. (2007) revealed a three-factor solution (*Prejudice, Appreciation,* and *Expectation*), with only 22 items included in the final version. The Mongolian (Gansukh et al., 2025) validation study described a one-factor solution with all items included, whereas the Hebrew version revealed a five-factor solution (*Prejudice, Appreciation, Expectation, Ambivalent,* and *Avoidance*) (Vitman-Schorr et al., 2014). Finally, the Greek study described a six-factor solution that appears to include only 20 items, although the reduction was not explicitly mentioned (Lambrinou et al., 2005). Such inconsistencies in reporting and item validation retention suggest that while the KAOP generally demonstrates structural robustness across cultural contexts, individual items may function differently depending on linguistic and cultural adaptation, highlighting the importance of transparent reporting of item-level decisions in validation studies.

Regarding the known-groups analysis, our study showed that all scales could significantly distinguish between gender, age groups, education, and attitudes toward older adults, with the exception of Factor 2 (gender and education) and KAOP-D-P (education). Although most studies confirm that older adults and women have a more positive attitude toward older adults (Zhang et al., 2024; Yáñez-Yáñez et al., 2022; Rush et al., 2017), there are findings that indicate our attitudes toward aging change as we grow older (Bodner et al., 2012). To our knowledge, only two validation studies performed *t-tests* on demographic variables. The study by Doherty et al. (2011) examined the KAOP scores by gender and found no significant differences. It is noteworthy that the male sample size was small (*n* = 19). Lambrinou et al. (2005) showed that variables such as gender and age do not affect attitudes toward older people. However, the mean age of their sample was 21 years, and 83% of the participants were female.

The convergent validity of the KAOP is supported by its significant associations with other measures of age-related attitudes. These measures include the ASD (Rosencranz and McNevin, 1969), the AS and FS scales (Kornadt et al., 2020). To our knowledge, only Ortiz-Rubio et al. (2020) also used the ASD in addition to the Positive and Negative Affect Schedule (PANAS) (Watson et al., 1988) and the Attitude-Older Adult and Aging-Visual Analog Scale (VAS) (Ligon et al., 2014) to examine convergent validity. Unexpectedly, they found no significant correlation with ASD. Nevertheless, our results indicate that the scale reliably captures both the negative and positive dimensions of attitudes toward older adults.

The internal consistency of the KAOP determined in the present study—with Cronbach’s *α* = 0.870 for the 34-item version and α = 0.874 for the 32-item version—can be considered good and falls within the upper range of values reported in the literature. Thus, the results are consistent with international validation studies, which also predominantly report reliability coefficients between α ≈ 0.80 and 0.90 (Alquwez et al., 2018; Erdemir et al., 2011; Küçükgüçlü et al., 2011; Fernández-Muñoz et al., 2023; Ogiwara et al., 2007; Yen et al., 2009; Vitman-Schorr et al., 2014; Iwasaki and Jones, 2008), underscoring the stability of internal consistency across different cultural contexts. At the same time, some studies report lower reliability coefficients (α ≈ 0.65–0.72) (Wanko Keutchafo and Kerr, 2024; Runkawatt et al., 2016; Abudu-Birresborn et al., 2023), suggesting possible influences from cultural differences, translation processes, or sample characteristics. This variation underscores that while the internal consistency of the KAOP is robust overall, it is not universally invariant across all contexts.

Despite the high reliability in the present study, the results should be interpreted in light of the known limitations of the KAOP. These include, in particular, the division into positively and negatively worded items, which is not always consistently replicated in empirical studies, with negatively worded items generally exhibiting higher α values (Kogan, 1961; Iwasaki and Jones, 2008; Vitman-Schorr et al., 2014; Matarese et al., 2013; Lambrinou et al., 2005). This was also evident in our study: for the negatively worded items, α = 0.826, and for the positively worded items, α = 0.784. Furthermore, the scale focuses on explicit attitudes, which means that implicit or behavioral aspects of ageism are not taken into account (Ortiz-Rubio et al., 2020). In addition, cultural differences in perceptions of aging and item interpretations may limit the comparability of results across different countries.

In the present study, the ICC for all versions of the scale was above 0.75, indicating good test–retest reliability across the testing intervals. For some subscales, such as the KAOP-D-P and Factor 2, the lower bounds fell below 0.75. This suggests that these subscales are slightly less stable and have more variability than the full scale. Overall, however, the findings provide strong evidence for the temporal stability of the KAOP-D. The revised 32-item version showed slightly improved reliability compared to the original scale. Similar values were reported in the Turkish (Küçükgüçlü et al., 2011; Erdemir et al., 2011), Iranian (Rejeh et al., 2012), and Chinese (Yen et al., 2009) validation studies after a 2- to 4-week interval. These results confirm that the KAOP is not only internally consistent but also measures reliably over time.

### 4.1 Strengths and limitations

This study has several notable strengths. First, it provides the first comprehensive translation, cultural adaptation, and psychometric validation of the KAOP for German-speaking populations, thereby addressing an important gap in the literature. This contribution enables both national research and cross-cultural comparisons of attitudes toward older adults.

Second, the relatively large sample size (*N* = 419) exceeds commonly recommended thresholds for psychometric analyses, enhancing the statistical power and robustness of the findings. While almost all other validation studies were conducted with nursing students, the participants in this study were members of the general population. In addition, the inclusion of a test–retest subsample (*n* = 211) over a four-week interval allowed for the assessment of temporal stability, strengthening the methodological rigor of the study. A further strength lies in the fact that the study goes beyond scale validation by examining associations with sociodemographic variables, contributing to a more nuanced understanding of attitudes toward older adults and their correlates.

Despite its strengths, this study has several limitations. First, the use of a convenience sample and online recruitment strategies may limit the generalizability of the findings. The sample appears to be relatively highly educated, which may not fully represent the broader German population.

Second, the cross-sectional design precludes any causal interpretations. Although differences across age groups were observed, longitudinal studies are needed to better understand changes in attitudes toward aging over time.

Third, all data were collected via self-report measures, which may introduce social desirability bias. Participants may have tended to report more positive attitudes toward older adults due to the socially sensitive nature of the topic.

Fourth, the removal of certain items (items 7 and 8) due to cultural inappropriateness indicates that some aspects of the original scale may not transfer seamlessly across cultural contexts. This highlights the need for caution when using the KAOP in cross-cultural research and suggests that item functioning may vary depending on cultural and linguistic settings.

Finally, structural validity was evaluated using PCA. Although this approach was chosen to facilitate comparison with previous KAOP validation studies, PCA is not specifically designed to identify latent constructs, and future research should examine the factor structure using exploratory factor analysis and confirm it with confirmatory factor analysis in an independent sample.

## 5 Conclusion

The KAOP-D-32 proved to be a robust instrument for measuring attitudes toward older adults, demonstrating high internal consistency and stability over time, as well as excellent content and construct validity. PCA identified two factors that do not align with the negative and positive statements, as shown by other validation studies. Convergent validity was supported by significant associations with related measures of age-related attitudes and perceptions, including the ASD, and the domains of the AS and the FS scales. These findings underscore the KAOP-D-32’s practical suitability for research on age-related attitudes.

## Data Availability

The datasets presented in this study can be found in online repositories. The names of the repository/repositories and accession number(s) can be found at: Open Science Framework: https://osf.io/8sae5/overview.
