# Supplementary material for: Psychometric properties of the Kogan Attitudes toward Older People Scale: evidence from a German general-population survey
Source: Front Psychol. 2026 Aug 25;17:1916164. doi: 10.3389/fpsyg.2026.1916164 (PMC13551729; doi:10.3389/fpsyg.2026.1916164)
Supplement: Supplementary file 2 [file Supplementary_File_2.docx]

Supplementary Material: Table1. The measure of sampling adequacy (MSA)

| 1 | .851 |
| --- | --- |
| 2 | .735 |
| 3 | .872 |
| 4 | .865 |
| 5 | .871 |
| 6 | .855 |
| 7 | .473 |
| 8 | .504 |
| 9 | .888 |
| 10 | .834 |
| 11 | .561 |
| 12 | .650 |
| 13 | .647 |
| 14 | .696 |
| 15 | .907 |
| 16 | .904 |
| 17 | .868 |
| 18 | .665 |
| 19 | .877 |
| 20 | .846 |
| 21 | .859 |
| 22 | .914 |
| 23 | .864 |
| 24 | .749 |
| 25 | .726 |
| 26 | .745 |
| 27 | .820 |
| 28 | .795 |
| 29 | .912 |
| 30 | .850 |
| 31 | .873 |
| 32 | .829 |
| 33 | .866 |
| 34 | .812 |
